# Efficacy and Cost-Efficacy of Biologic Therapies for Moderate to Severe Psoriasis: A Meta-Analysis and Cost-Efficacy Analysis Using the Intention-to-Treat Principle

**DOI:** 10.1155/2014/862851

**Published:** 2014-01-29

**Authors:** Ching-Chi Chi, Shu-Hui Wang

**Affiliations:** ^1^Department of Dermatology and Centre for Evidence-Based Medicine, Chang Gung Memorial Hospital, Chiayi 61363, Taiwan; ^2^College of Medicine, Chang Gung University, Taoyuan 33302, Taiwan; ^3^Department of Dermatology, Far Eastern Memorial Hospital, Banciao, New Taipei 22060, Taiwan; ^4^Oriental Institute of Technology, New Taipei 22061, Taiwan

## Abstract

*Background*. Compared to conventional therapies, biologics are more effective but expensive in treating psoriasis. *Objective*. To evaluate the efficacy and cost-efficacy of biologic therapies for psoriasis. *Methods*. We conducted a meta-analysis to calculate the efficacy of etanercept, adalimumab, infliximab, and ustekinumab for at least 75% reduction in the Psoriasis Area and Severity Index score (PASI 75) and Physician's Global Assessment clear/minimal (PGA 0/1). The cost-efficacy was assessed by calculating the incremental cost-effectiveness ratio (ICER) per subject achieving PASI 75 and PGA 0/1. *Results*. The incremental efficacy regarding PASI 75 was 55% (95% confidence interval (95% CI) 38%–72%), 63% (95% CI 59%–67%), 71% (95% CI 67%–76%), 67% (95% CI 62%–73%), and 72% (95% CI 68%–75%) for etanercept, adalimumab, infliximab, and ustekinumab 45 mg and 90 mg, respectively. The corresponding 6-month ICER regarding PASI 75 was $32,643 (best case $24,936; worst case $47,246), $21,315 (best case $20,043; worst case $22,760), $27,782 (best case $25,954; worst case $29,440), $25,055 (best case $22,996; worst case $27,075), and $46,630 (best case $44,765; worst case $49,373), respectively. The results regarding PGA 0/1 were similar. *Conclusions*. Infliximab and ustekinumab 90 mg had the highest efficacy. Meanwhile, adalimumab had the best cost-efficacy, followed by ustekinumab 45 mg and infliximab.

## 1. Background

Psoriasis is a chronic inflammatory disease affecting 1–3% of the general population and incurs a considerable economic burden [1]. A number of biologics have been introduced for treating moderate to severe psoriasis. Etanercept is a fusion protein that binds to and neutralizes tumor necrosis factor (TNF) [2]. Adalimumab is a recombinant monoclonal antibody that binds to TNF and blocks its interaction from TNF receptors [3]. Infliximab is a chimeric monoclonal antibody which binds and neutralizes TNF [4]. Ustekinumab is a monoclonal antibody against the p40 subunit of the IL-12 and IL-23 cytokines which are involved in inflammatory and immune responses [5]. Short-term trials on these biologics showed that 47%–88% of the participants achieved at least 75% reduction in the Psoriasis Area and Severity Index score (PASI 75) after treatment for 10 to 16 weeks [2–5].

Biologics therapies for psoriasis are expensive. Based on the US drug price in April 2010 [6], the 6 month drug costs are $17,954, $13,429, $19,725, $16,787, and $33,574 for the etanercept, adalimumab, infliximab (for a person weighing 81–100 kg), ustekinumab 45 mg, and ustekinumab 90 mg regimens approved by the Food and Drug Administration (FDA) (see below), respectively. Healthcare payers therefore often have an eligibility criterion for the reimbursement of biologics therapies. Patients with moderate to severe psoriasis (defined as involvement of greater than 5% body surface area or involvement of ≤5% body surface area affecting sensitive areas or areas that significantly impact daily function (e.g., palms, soles, head, neck, or genitalia) are eligible for reimbursement if the psoriasis has not responded to phototherapy and systemic agents (such as acitretin, methotrexate, and cyclosporine) or if the patients are intolerant of, or have a contraindication to, these treatments [7].

The drug costs for treating psoriasis in the US have increased by 30% from 2000 to 2008, with a major contribution from biologics [1]. The increasing drug spending leads to an economic burden of healthcare systems. The objective of this study was to use the best evidence to assess the efficacy and cost-efficacy of biologic therapies for treating moderate to severe psoriasis. It is our hope that this will assist in efficient allocation of limited resources in treating psoriasis. We did not analyze conventional therapies in this study as biologic therapies are primarily used as second-line treatments when conventional therapies fail or are contraindicated.

## 2. Methods

### 2.1. Meta-Analysis

We performed a meta-analysis of randomized controlled trials using the intention-to-treat (ITT) principle to assess the efficacy of etanercept, adalimumab, infliximab, and ustekinumab in treating psoriasis. We searched the Cochrane Central Register of Controlled Trials, MEDLINE, and EMBASE for relevant studies on November 23, 2012. The inclusion criteria of studies were randomized placebo-controlled trials which assessed the efficacy of etanercept, adalimumab, infliximab, and ustekinumab in treating moderate to severe psoriasis in adults by using the FDA-approved regimens for at least 6 months.

We included trials that adhered to the regimens approved by the US FDA. Trials that did not use an approved regimen were excluded. If a multiarm placebo-controlled trial contained an arm using an approved regimen and another using an unapproved regimen, we extracted relevant data from the arm using the approved regimen and the placebo arm. The approved etanercept regimen for treating psoriasis is 50 mg twice weekly in the first 12 weeks, followed by 50 mg once weekly or 25 mg twice weekly [2]. The approved adalimumab regimen is 80 mg at week 0, followed by 40 mg every other week [3]. The approved infliximab regimen is 5 mg/kg administered at weeks 0, 2, and 6, followed by 5 mg/kg every 8 weeks thereafter [4]. The approved ustekinumab regimen is 45 mg (or 90 mg for patients weighing over 100 kg) at week 0 and week 4, followed by 45 mg (or 90 mg for patients weighing over 100 kg) every 12 weeks [5].

**Figure 1:**
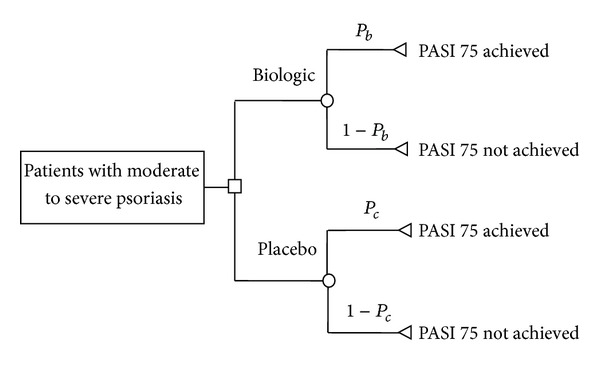
Decision tree.

The primary efficacy outcome was the proportion of participants achieving PASI 75 at month 6 (week 24–28 were acceptable). We built a decision tree for analysis as shown in Figure 1. The proportion of participants achieving PASI 75 was *P*_*b*_ and *P*_*c*_ in the biologics and placebo groups, respectively. The secondary efficacy outcome was the proportion of participants achieving Physician's Global Assessment clear or minimal (PGA 0/1) at month 6. We calculated the outcomes based on all randomized participants, that is, ITT analysis. All randomized participants with missing outcome data were considered treatment failure. If a trial did not have data on PASI 75 and PGA 0/1 response after 6 months' use of placebo because the placebo groups were switched to biologics treatment before month 6, we used the last observation carried forward approach to estimate the outcomes. For example, if the placebo group was switched to biologic treatments at week 12, we used the PASI 75 and PGA 0/1 data at week 12 as the estimated efficacy after 6 months' use of placebo.

We defined incremental efficacy as the absolute increase in the proportion of participants achieving a prespecified outcome after a biologic therapy when compared to placebo, that is, *P*_*b*_ − *P*_*c*_ (see Figure 1). We calculated the 6-month incremental efficacy of each biologic regimen for PASI 75 and PGA 0/1 response, respectively. When more than one trial were available for an outcome, we applied a meta-analysis technique to calculate the pooled efficacy and 95% confidence interval (CI) by using the DerSimonian and Laird random-effects model [8]. The Review Manager 5.1 (Nordic Cochrane Centre, Cochrane Collaboration, Copenhagen, Denmark, 2011) was used for meta-analysis.

### 2.2. Cost-Efficacy Analysis

For cost-efficacy analysis, we considered the direct drug costs of the approved regimen based on the US drug price in April 2010 [6]. The direct costs are Cost_*b*_ and Cost_*c*_ in the biologics and placebo groups, respectively. The costs of placebo were assumed to be nil. For infliximab, we assumed a patient bodyweight of 81–100 kg and wasting of remaining vial after use (one vial contains 100 mg infliximab). We assessed the cost-efficacy by calculating the incremental cost-effectiveness ratio (ICER), which was the ratio of the increase in costs to the efficacy, that is, (Cost_*b*_ − Cost_c_)/(*P*_*b*_ − *P*_*c*_). In other words, the ICER was the average cost for one participant to achieve a prespecified outcome. The lower the ICER was, the more cost-effective a biologic therapy was.

We calculated the 6-month (24 weeks) base case ICERs of each biologic therapy according to the incremental efficacy when compared to placebo in terms of PASI 75 and PGA 0/1. We also calculated the worst and best case ICERs based on the lower and upper 95% confidence limits of the incremental efficacy, respectively. The range between the worst and best case ICERs can be regarded as the 95% CI of the ICER. In addition, we used the base case data to conduct an incremental analysis after excluding the least cost-effective biologic therapy and calculated the incremental costs per additional PASI 75 or PGA 0/1 responder between the remaining biologic therapies.

## 3. Results

### 3.1. Meta-Analysis

**Figure 2:**
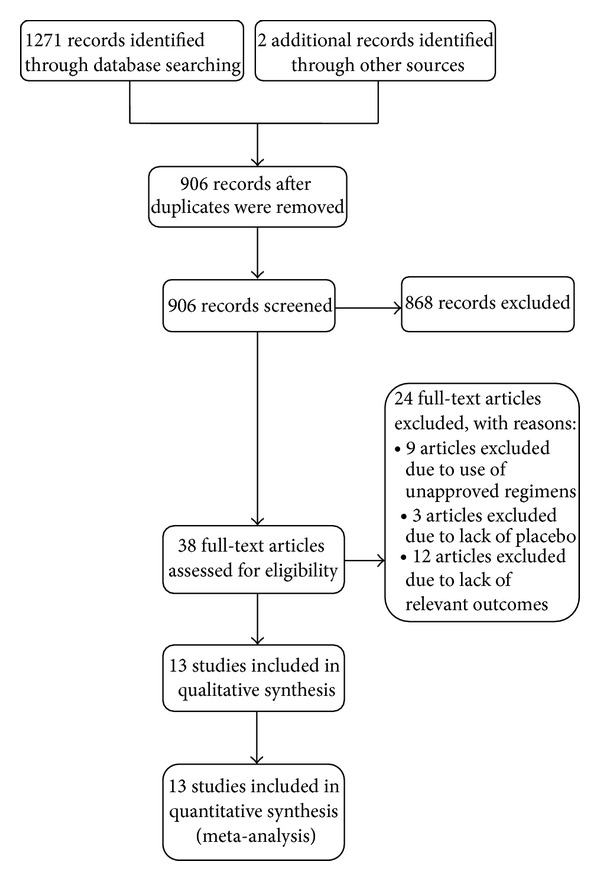
Study flow diagram.

**Figure 3:**
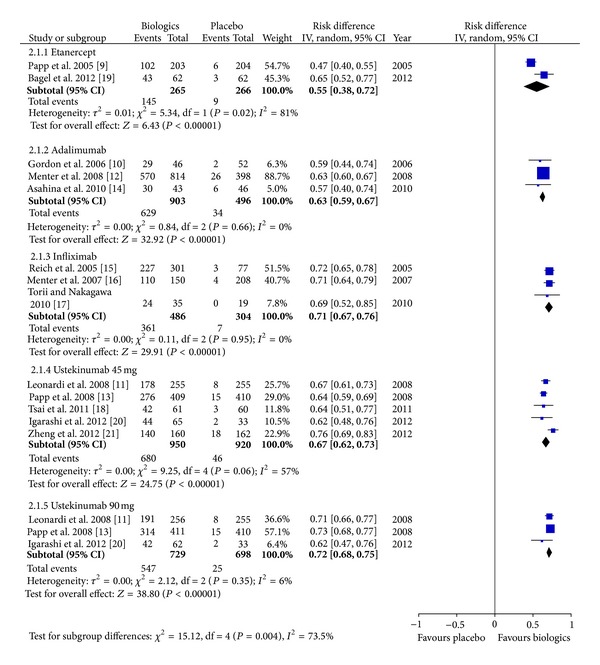
Meta-analysis based on at least 75% reduction in the Psoriasis Area and Severity Index score.

**Figure 4:**
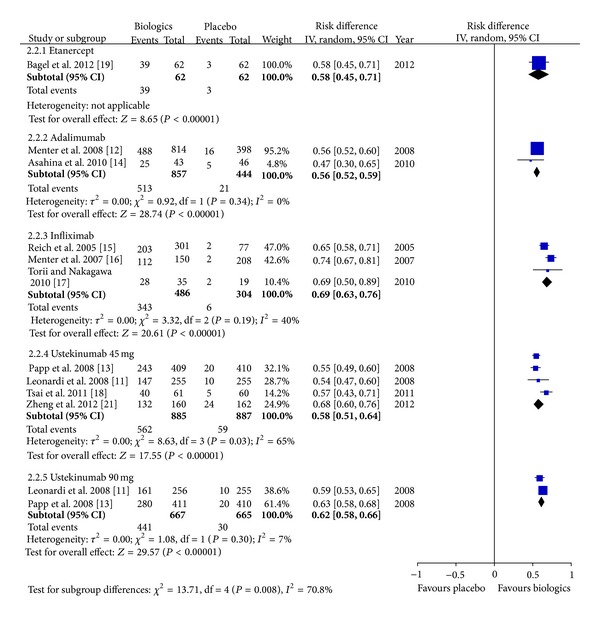
Meta-analysis based on Physician's Global Assessment clear or almost clear.

As shown in Figure 2, 1271 records were identified through searching the databases and 2 additional records were obtained from a pharmaceutical company. After removal of duplicates and exclusion due to use of unapproved regimens, lack of placebo, or relevant outcomes, 13 trials with a total of 5309 participants were included [9–21]. All the included trials were of high quality when appraised by using the Cochrane Collaboration's tool for assessing risk of bias in randomized trials [8]. The efficacy outcomes of the included trials are summarized in Table 1. The meta-analysis (Figure 3) found that the pooled incremental efficacy of PASI 75 response was 55% (95% CI 38%–72%), 63% (95% CI 59%–67%), 71% (95% CI 67%–76%), 67% (95% CI 62%–73%), and 72% (95% CI 68%–75%) for etanercept, adalimumab, infliximab, ustekinumab 45 mg, and ustekinumab 90 mg, respectively. The pooled incremental efficacy of PGA 0/1 response was 58% (95% CI 45%–71%), 56% (95% CI 52%–59%), 69% (95% CI 63%–76%), 58% (95% CI 51%–64%), and 62% (95% CI 58%–66%) for etanercept, adalimumab, infliximab, ustekinumab 45 mg, and ustekinumab 90 mg, respectively (Figure 4).

### 3.2. Cost-Efficacy

Based on the ICER as to PASI 75 response (Table 2), adalimumab had the best cost-efficacy ($21,315 in the base case, $20,043 in the best case, and $22,760 in the worst case), followed by ustekinumab 45 mg ($25,055 in the base case, $22,996 in the best case, and $27,075 in the worst case) and infliximab ($27,782 in the base case, $25,954 in the best case, and $29,440 in the worst case). For etanercept, the 6-mo ICER was $32,643 in the base case, $24,936 in the best case, and $47,246 in the worst case. Ustekinumab 90 mg had the highest 6-mo ICER ($46,630 in the base case, $44,765 in the best case, and $49,373 in the worst case).

Based on PGA 0/1 response (Table 3), adalimumab had the most favorable 6-month ICER (adalimumab: $23,980 in the base case, $22,760 in the best case, and $25,824 in the worst case), followed by infliximab ($28,587 in the base case, $25,954 in the best case, and $31,310 in the worst case) and ustekinumab 45 mg ($28,943 in the base case, $26,229 in the best case, and $32,915 in the worst case). Etanercept had a wide 95% CI of a 6-month ICER (base case $30,954; best case $25,287; worst case $39,897) and overlapped with adalimumab and ustekinumab 45 mg. Ustekinumab 90 mg had the highest 6-month ICER of PGA 0/1 (base case $54,151; best case $50,869; worst case $57,886).

The base case incremental analysis (Table 4) showed when considering PASI 75 response, etanercept was dominated. Adalimumab was likely to be the most cost-effective, with a cost of $21,315 per PASI 75 responder when compared to placebo. Ustekinumab 45 mg had a cost of $83,950 per additional PASI 75 responder when compared to adalimumab, while infliximab had a cost of $68,175 per additional PASI 75 responder when compared to ustekinumab 45 mg. Ustekinumab 90 mg had a cost of $1,384,900 per additional PASI 75 responder when compared to infliximab. On the other hand, when considering PGA 0/1 response, adalimumab was likely to be the most cost-effective biologic therapy, with a cost of $23,980 per PASI 75 responder when compared to placebo. Ustekinumab 45 mg had a cost of $167,900 per additional PGA 0/1 responder when compared with adalimumab. Compared to ustekinumab 45 mg, ustekinumab 90 mg and infliximab had a cost of $3,358,150 and $26,709 per additional PGA 0/1 responder, respectively.

## 4. Discussion

Psoriasis is a chronic dermatosis which cannot be cured and imposes an impact on quality of life comparable to that experienced by patients with type 2 diabetes mellitus or chronic lung disease [22]. Clinical efficacy and cost-efficacy are thus important in allocating limited resources for treatments. The present study assessed the 6-month efficacy and cost-efficacy of biologic therapies by examining two outcomes (PASI 75 and PGA 0/1), and can serve as a useful reference for dermatologists and policy makers. Our meta-analysis revealed that infliximab and ustekinumab 90 mg had a higher pooled incremental efficacy as to either PASI 75 or PGA 0/1 when compared to other biologics. On the other hand, adalimumab had the best cost-efficacy based on either PASI 75 or PGA 0/1, followed by ustekinumab 45 mg and infliximab. Etanercept had a wide range of cost-efficacy estimate due to limited available data and was dominated in the incremental analysis. Ustekinumab 90 mg had very high costs of $1,384,900 and $3,358,150 per additional PASI 75 and PGA 0/1 responders when compared to the next best regimen, which were above any known conventional willingness to pay threshold.

Previous economic analyses on biologics for treating psoriasis determined the efficacy based on data from short-term endpoints at weeks 10 to 16 [23–27]. However, the efficacy may differ with time. For example, the proportion of PASI 75 responders to etanercept increased from 59% at week 12 to 69% at week 24 [19]. The proportion of PASI 75 responders to adalimumab increased from 53% at week 12 to 64% at week 24 [10]. By using a 6-month data, our study provides a reliable reference as to intermediate-term efficacy and cost-efficacy. We originally planned to collect efficacy outcome assessed at week 52, but could not obtain relevant data because of the fact that the length of trial was less than 52 weeks [9], discontinuation of biologic therapy for participants with inadequate response [20], and rerandomization of participants with sustained PASI 75 response to either placebo or biologics [11–13].

Previous economic analyses only included efficacy data from trials conducted in the US and Europe where most participants were Caucasians [23–27]. The present analysis included efficacy data from four Asian trials [14, 17, 18, 20, 21], and thus it has a better generalizability in a multiethnic setting like the US.

Many trials included in this study used modified ITT analysis to assess efficacy outcomes, that is, inclusion of randomized subjects who received at least one dose of the study drug in statistical analyses [9, 10]. When assessing efficacy outcomes at month 6, some trials used perprotocol analysis, that is, only including subjects who stayed in the trials in analysis [11, 13, 15–18, 20, 21]. Both approaches excluded those lost to follow-up due to lack of efficacy from statistical analyses, which may lead to biased efficacy estimates [8]. In our meta-analysis, we recalculated all efficacy outcome data by using the ITT approach, that is, we included all randomized subjects in statistical analyses and considered those subjects with missing data as treatment failure. Therefore, our meta-analysis provides less biased efficacy estimates and best mimics actual practice where patients are able to drop out of treatment and change treatment groups.

Similar to previous economic analyses [25, 27], the present cost-efficacy analysis only considered drug costs. Other costs for administering biologics and indirect costs were not considered. The cost efficacy of infliximab will decrease if the indirect cost and the time missed from work due to intravenous administration are considered.

Biologic therapies are generally conceived to be expensive when compared to conventional therapies. However, a study revealed that introduction of biologics therapies reduced the total healthcare costs for patients who previously required long-term hospitalization for disease control, as hospitalizations were shortened or no longer needed [28]. Therefore, careful selection of patients appropriate for biologic therapies may be cost-saving on the ground of avoidance of ineffective conventional treatments, reduction of hospitalization costs, increased productivity, and reduction of indirect costs.

Although drug costs are an important concern in choosing biologics, they are not the sole determinant. Patients' unique values and circumstances should be considered in decision making [29]. For example, the total number of injections in the first 6 months' therapy is 36, 13, 5, and 3 for etanercept, adalimumab, infliximab, and ustekinumab, respectively. Patients who are afraid of injections or dislike the injection pain may prefer ustekinumab therapy.

Presence of concomitant psoriatic arthritis may affect the choice of biologics. Anti-TNF*α* agents (i.e., etanercept, adalimumab, and infliximab) have established efficacy in treating psoriatic arthritis [30] and are therefore preferred in patients with concomitant psoriatic arthritis. A trial found that ustekinumab reduced symptoms and signs of psoriatic arthritis, but the administered regimen (ustekinumab 90 mg or 63 mg every week for 4 weeks) differed from those used in treating psoriasis [31]. Another randomized trial found that ustekinumab administered using the approved regimens improved joint pain visual analogue scale, but the efficacy appeared varying and lacked a dose-response relationship [20].

## 5. Conclusions

Infliximab and ustekinumab 90 mg had a higher pooled efficacy as to either PASI 75 or PGA 0/1 when compared to other biologics. On the other hand, adalimumab had the lowest average costs per patient achieving PASI 75 or PGA 0/1 response, followed by ustekinumab 45 mg and infliximab. Etanercept and ustekinumab 90 mg had an unfavorable cost-efficacy. Clinicians and policy-makers should consider the efficacy and cost-efficacy evidence along with patients' values and characteristics (such as presence of psoriatic arthritis) in deciding how to efficiently allocate resources in treating psoriasis.

## Figures and Tables

**Table 1:** Efficacy outcomes of included trials.

Trial (first author, publication year)	Interventions (only comparisons relevant to this meta-analysis listed)	Randomised participants (*n*)	Timing of outcome assessment	PASI 75 [*n* (%)]	PGA 0/1 [*n* (%)]
*Etanercept trials *					

Papp et al., 2005 [9]	Intervention:				
	Etanercept 50 mg BIW for 12 weeks, then 25 mg BIW for 12 weeks.	203	Week 24	102 (50%)	NA
	Control intervention:				
	Placebo BIW for 12 weeks, then 25 mg BIW for 12 weeks.	204	Week 12	6 (3%)	NA

Bagel et al., 2012 [19]	Intervention:				
	Etanercept 50 mg BIW for 12 weeks, then 50 mg QW for 12 weeks.	62	Week 24	43 (69%)	39 (63%)
	Control intervention:				
	Placebo BIW for 12 weeks, then 50 mg BIW for 12 weeks.	62	Week 12	3 (5%)	3 (5%)

*Adalimumab trials *					

Gordon et al., 2006 [10]	Intervention:				
	Adalimumab 80 mg at week 0, then 40 mg EOW starting at week 1.	46	Week 24	29 (63%)	29 (63%)
	Control intervention:				
	Placebo QW, then adalimumab 80 mg at week 12, followed by 40 mg EOW from week 13 on.	52	Week 12	2 (4%)	NA

Menter et al., 2008 [12]	Intervention:				
	Adalimumab 80 mg at week 0, then 40 mg EOW starting at week 1.	814	Week 24	570 (70%)	488 (60%)
	Control intervention:				
	Placebo QW at week 0, then EOW beginning *t* week 1 and through week 15, followed by 40 mg EOW from week 16 on.	398	Week 16	26 (7%)	16 (4%)

Asahina et al., 2010 [14]	Intervention:				
	Adalimumab 80 mg at week 0, then 40 mg EOW starting at week 2.	43	Week 24	30 (70%)	25 (58%)
	Control intervention:				
	Placebo EOW for 24 weeks.	46	Week 24	6 (13%)	5 (11%)

*Infliximab trials *					

Reich et al., 2005 [15]	Intervention:				
	Infliximab 5 mg/kg at weeks 0, 2, and 6, then every 8 weeks.	301	Week 24	227 (75.4%)	203 (67.4%)
	Control intervention:				
	Placebo at weeks 0, 2, and 6, then every 8 weeks.	77	Week 24	3 (3.8%)	2 (2.6%)

Menter et al., 2007 [16]	Intervention:				
	Infliximab 5 mg/kg at weeks 0, 2, and 6, then every 8 week.	150	Week 26	110 (73.3%)	112 (74.6%)
	Control intervention				
	Placebo at weeks 0, 2, and 6, switched to infliximab 5 mg/kg at week 16.	208	Week 10	4 (1.9%)	2 (1%)

Torii and Nakagawa 2010 [17]	Intervention:				
	Infliximab 5 mg/kg at weeks 0, 2, and 6, then every 8 weeks.	35	Week 26	24 (68.6%)	28 (80%)
	Control intervention: Placebo at weeks 0, 2, and 6, switched to infliximab 5 mg/kg at weeks 16.	19	Week 10	0 (0%)	2 (10.5%)

*Ustekinumab trials *					

Leonardi et al., 2008 [11]	Intervention:				
	(i) Ustekinumab 45 mg at weeks 0 and 4, then every 12 weeks.	255	Week 28	178 (71%)	147 (58%)
	(ii) Ustekinumab 90 mg at weeks 0 and 4, then every 12 weeks.	256	Week 28	191 (75%)	161 (63%)
	Control intervention:				
	Placebo at weeks 0 and 4, then ustekinumab 45 or 90 mg every 12 weeks.	255	Week 12	8 (3%)	10 (4%)

Papp et al., 2008 [13]	Intervention:				
	(i) Ustekinumab 45 mg at weeks 0 and 4, then every 12 weeks.	409	Week 28	276 (67%)	243 (59%)
	(ii) Ustekinumab 90 mg at weeks 0 and 4, then every 12 weeks.	411	Week 28	314 (76%)	280 (68%)
	Control intervention:				
	Placebo at weeks 0 and 4, then ustekinumab 45 or 90 mg every 12 weeks.	410	Week 12	15 (4%)	20 (5%)

Tsai et al., 2011 [18]	Intervention:				
	Ustekinumab 45 mg at weeks 0 and 4, then every 12 weeks.	61	Week 28	42 (69%)	40 (66%)
	Control intervention:				
	Placebo at weeks 0 and 4, then ustekinumab 45 or 90 mg every 12 weeks.	60	Week 12	3 (5%)	5 (8%)

Igarashi et al., 2012 [20]	Intervention:				
	(i) Ustekinumab 45 mg at weeks 0 and 4, then every 12 weeks.	65	Week 28	44 (68%)	NA
	(ii) Ustekinumab 90 mg at weeks 0 and 4, then every 12 weeks.	62	Week 28	42 (68%)	NA
	Control intervention:				
	Placebo at weeks 0 and 4, then ustekinumab 45 or 90 mg every 12 weeks.	33	Week 12	2 (6%)	3 (9%)

Zheng et al., 2012 [21]	Intervention:				
	Ustekinumab 45 mg at weeks 0 and 4, then every 12 weeks.	160	Week 28	140 (88%)	132 (83%)
	Control intervention:				
	Placebo at weeks 0 and 4, then ustekinumab 45 or 90 mg every 12 weeks.	162	Week 12	18 (11%)	24 (15%)

BIW: twice weekly; EOW: every other week, NA: not available; PASI 75: at least 75% reduction in the Psoriasis Area and Severity Index score; PGA 0/1: Physician's Global Assessment clear or almost clear; QW: once weekly.

**Table 2:** Incremental efficacy and cost-efficacy based on at least 75% reduction in the Psoriasis Area and Severity Index score.

Biologics	Pooled incremental efficacy			6-month incremental cost-effectiveness ratio		
	Base case	Best case	Worst case	Base case	Best case	Worst case
Etanercept	55%	72%	38%	$32,643	$24,936	$47,246
Adalimumab	63%	67%	59%	$21,315	$20,043	$22,760
Infliximab	71%	76%	67%	$27,782	$25,954	$29,440
Ustekinumab 45 mg	67%	73%	62%	$25,055	$22,996	$27,075
Ustekinumab 90 mg	72%	75%	68%	$46,630	$44,765	$49,373

**Table 3:** Incremental efficacy and cost-efficacy based on Physician's Global Assessment clear or almost clear.

Biologics	Pooled incremental efficacy			6-month incremental cost-effectiveness ratio		
	Base case	Best case	Worst case	Base case	Best case	Worst case
Etanercept	58%	71%	45%	$30,954	$25,287	$39,897
Adalimumab	56%	59%	52%	$23,980	$22,760	$25,824
Infliximab	69%	76%	63%	$28,587	$25,954	$31,310
Ustekinumab 45 mg	58%	64%	51%	$28,943	$26,229	$32,915
Ustekinumab 90 mg	62%	66%	58%	$54,151	$50,869	$57,886

**Table 4:** Base case incremental analysis.

	6-month costs	Incremental efficacy of PASI 75 response	ICER (costs per additional PASI 75 responder)
Etanercept	$17,954	55%	Dominated
Adalimumab	$13,429	63%	$21,315^a^
Ustekinumab 45 mg	$16,787	67%	$83,950^b^
Infliximab	$19,725	71%	$68,175^c^
Ustekinumab 90 mg	$33,574	72%	$1,384,900^d^

		Incremental efficacy of PGA 0/1 response	ICER (costs per additional PGA 0/1 responder)

Adalimumab	$13,429	56%	$23,980^a^
Ustekinumab 45 mg	$16,787	58%	$167,900^b^
Etanercept	$17,954	58%	Dominated
Ustekinumab 90 mg	$33,574	62%	$3,358,150^c^
Infliximab	$19,725	69%	$26,709^c^

^a^Compared to placebo.

^b^Compared to adalimumab.

^c^Compared to ustekinumab 45 mg.

^d^Compared to infliximab.
